# Case report: An incidental finding of a left-sided supernumerary kidney

**DOI:** 10.3389/fmed.2024.1490211

**Published:** 2025-01-17

**Authors:** Aklilu Getachew, Nathan K. Suga, Jochebed K. Suga, Nuhamin D. Kiflemariam

**Affiliations:** Department of Surgery, Myungsung Medical College/MCM Comprehensive Specialized Hospital, Addis Ababa, Ethiopia

**Keywords:** supernumerary kidney, congenital anomalies, malrotated kidney, Ethiopia, CT urography

## Abstract

A supernumerary kidney is an extremely rare congenital renal anomaly that is characterized by the presence of one or more extra kidneys. Unilateral cases occur more commonly on the left side. Reporting such uncommon anomalies is important for several reasons, such as improving diagnosis and treatment, educating clinicians and radiologists about the identification and treatment of supernumerary kidneys, and comparing the case to existing literature to highlight similarities and differences in presentation, management, or outcomes. A 35-year-old male patient presented to our hospital in Addis Ababa, Ethiopia, with left lower flank pain. His blood pressure was elevated during the initial visit; however, the results of the physical examination and laboratory investigations were unremarkable. Abdominopelvic ultrasound and computed tomographic (CT) urography confirmed the diagnosis of a left-sided supernumerary kidney, with no associated abnormalities. In such cases, the diagnosis of a supernumerary kidney is made using an abdominal ultrasound scan, intravenous urography (IVU), CT urography, and magnetic resonance imaging (MRI). Treatment depends on the patient’s symptoms. Asymptomatic cases must be followed up regularly. If a supernumerary kidney is nonfunctional or associated with other abnormalities, a nephrectomy is indicated. We treated our patient with adequate analgesia and scheduled a follow-up.

## 1 Introduction

A supernumerary kidney is an extremely rare congenital renal anomaly, characterized by the presence of one or more extra kidneys (1). Cases of true supernumerary kidneys are considered one of the rarest of all pathological renal conditions, with approximately 100 cases reported in the literature. A bilateral supernumerary kidney is an even rarer anomaly, with only four cases reported to date. For the first time, Voigtel made an intriguing and succinct mention of this topic in 1805 (2, 3). Unilateral cases occur more commonly on the left side (4). In the past, the majority of supernumerary kidney cases were discovered following surgery or autopsy; however, nowadays, even incidental cases can be detected using various imaging modalities (5). Reporting these rare anomalies is important for several reasons, such as improving diagnosis and treatment, educating clinicians and radiologists about the identification and treatment of supernumerary kidneys, and comparing the case to existing literature to highlight similarities and differences in presentation, management, or outcomes. We report an incidental finding of a left-sided supernumerary kidney.

## 2 Case presentation

**Figure 1:**
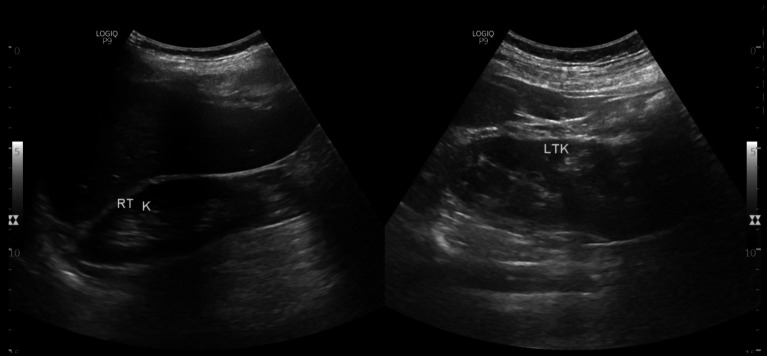
Abdominal ultrasonogram showing right side single kidney and left sided native and supernumerary kidneys.

**Figure 2:**
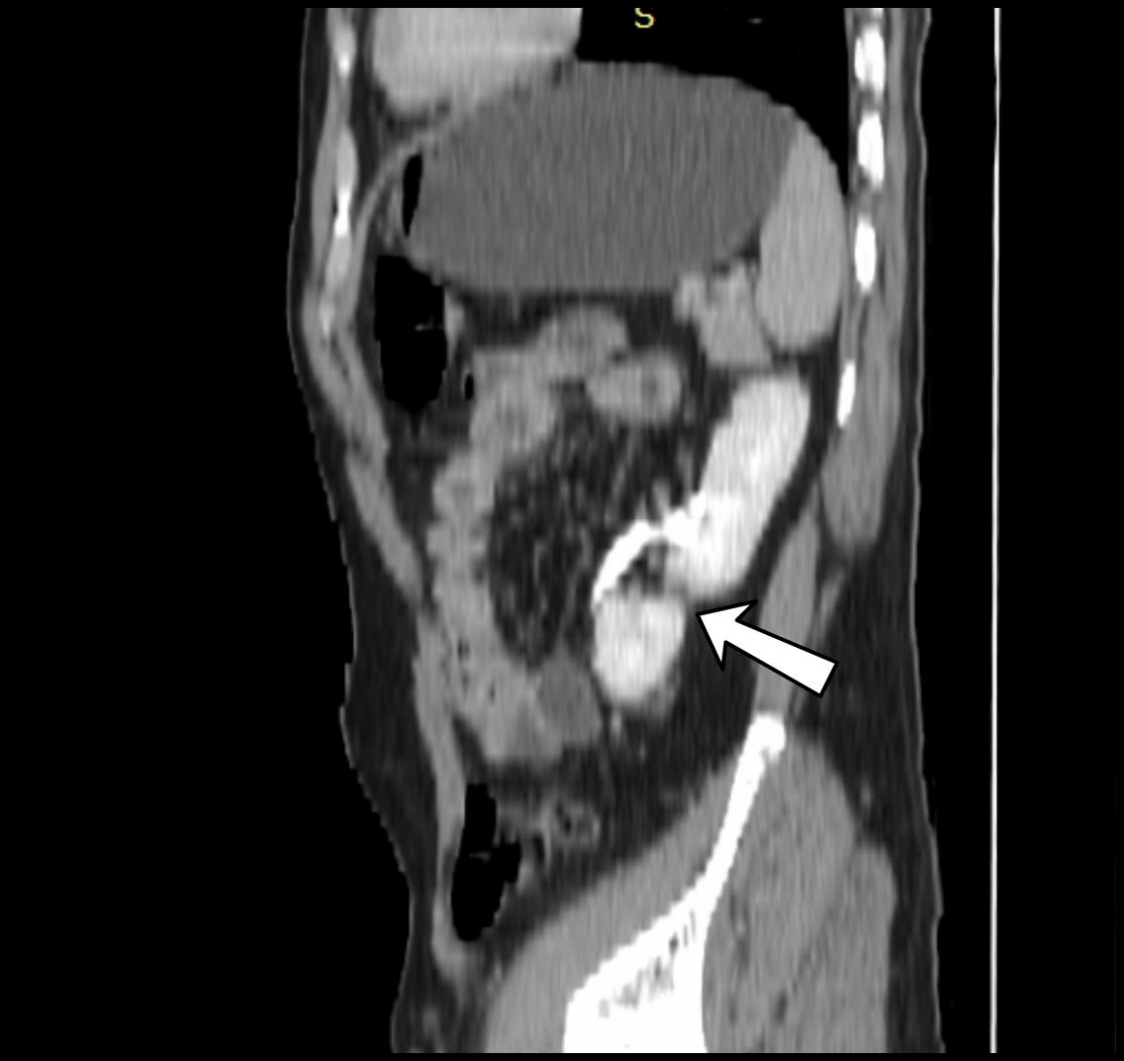
Sagittal view of left elongated and malrotated native kidney during the nephrogenic phase of CT urography, in conjunction with a supernumerary kidney linked to it at the lower pole.

**Figure 3:**
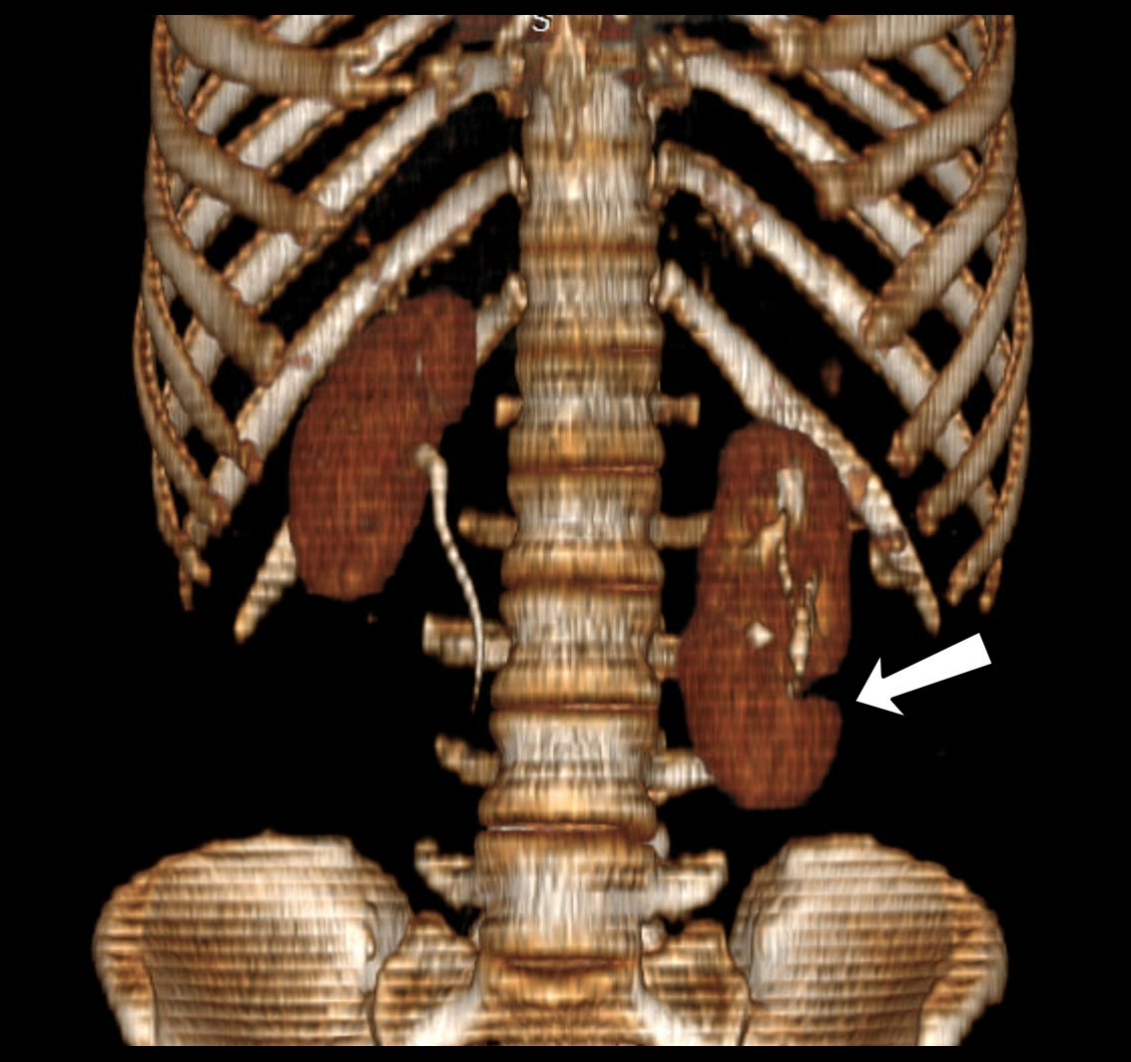
3D reconstruction of the coronal view of CT urography showing left sided native and supernumerary kidneys extending up to L4.

A 35-year-old male patient presented to our hospital in Addis Ababa, Ethiopia, with left lower flank pain. The patient experienced dull-aching pain that did not radiate. His blood pressure was elevated to the level of 160/90 mmHg during the first visit; however, the results of the physical examination were unremarkable. The results of renal function tests and urinalysis were within the normal range. An abdominal ultrasound showed that the right kidney was of normal size and shape with a smooth contour and normal cortical echotexture. A left supernumerary fused kidney with normal corticomedullary differentiation was also visualized. No stone, mass, or hydronephrosis was evident. Two separate renal arteries (both arising from the aorta) and veins were observed. A computed tomographic (CT) urography was then performed. Non-contrast, nephrogenic phase, cortico-medullary phase, and delayed phase images were obtained from the lung bases to the symphysis pubis. Then, a three-dimensional (3D) digital reconstruction was performed, and it showed an elongated and malrotated left kidney, with the hilum facing posterolaterally, and a fused, smaller supernumerary kidney measuring 6.4 cm at its lower pole. The native and fused kidneys together measured 13 cm in the long axis, and they formed a single pelvic and ureteric system with no hydronephrosis. All renal units showed good contrast uptake and timely excretion. After receiving adequate analgesics, the patient was scheduled for follow-up, and his blood pressure was normalized during the subsequent visits (Figures 1–3).

## 3 Discussion

A supernumerary kidney is an extra kidney in addition to the two kidneys that are typically present. It may or may not be fused to the other kidneys (6). Less than 100 cases of this rare congenital anomaly have been reported (7). The maximum number of supernumerary kidneys reported in the literature is four. Embryologically, supernumerary kidneys are formed by aberrant division of the nephrogenic cord into two metanephric blastemas, with bifurcation of one bud (3). This anomaly develops around the fifth to seventh week of gestation when urogenital system development occurs (8). It is important to distinguish a supernumerary kidney from the more typical duplex kidney, which is characterized by two pelvicalyceal systems connected by a single ureter or double ureter (9). A parenchymal bridge or fibrous tissue may connect a supernumerary kidney to the ipsilateral kidney, or it may be completely detached, with its own blood supply and capsule (10). A supernumerary kidney is often functional and typically smaller than the native kidney (11). The vascular distribution of the supernumerary kidney is highly variable, and no consistent pattern of arterial origin or venous outflow has been identified to date. Arterial and venous branches from the common iliac, internal iliac, and even mesenteric vessels have been reported (12). There are two different kinds of supernumerary kidneys: those draining through a bifid ureter and those draining through a separate ureter. In our case, the patient had a single ureteric system shared by the two kidneys on the left side. The supernumerary kidney is positioned cranially relative to the normal kidney when a separate ureter is visible; when a bifid system is present, the supernumerary kidney is found to be located caudally (5). In the majority of cases, men are affected by this anomaly. Among them, the majority of cases are unilateral and occur more commonly on the left side (8). Conjunctive anomalies associated with supernumerary kidneys have been documented, despite the extremely low incidence of this anomaly. These include horseshoe kidney malformation, ventricular septal defects, neural tube defects, and cloacal anomalies, such as urethral atresia, vaginal atresia, ectopic ureter implantation, imperforate anus and duplication of the penile urethra (13). Our patient did not have any additional conjunctive malformations or defects associated with the supernumerary kidney.

Although this anomaly is usually asymptomatic, symptoms may include fever, hypertension, abdominal discomfort, or a palpable mass. Several pathological conditions, including hydronephrosis, pyelonephritis, pyonephrosis, renal and ureteral calculi, and malignant and benign neoplasms, may affect the renal moieties in patients with a supernumerary kidney (6). The diagnostic options for the supernumerary kidney include an abdominal ultrasound scan, intravenous urography (IVU), CT urography, and magnetic resonance imaging (MRI). The type of collecting system, the blood supply to the supernumerary kidney, and any accompanying anomalies can all be determined with the help of IVU, CT, and MRI (4). Inaccuracies in the evaluation of diagnostic procedures that fail to identify supernumerary kidneys appear to be a significant contributing factor to increased morbidity. This may result in unnecessary interventions, such as biopsies or surgery, which may cause more complications (5).

Treatment depends on the patient’s symptoms. Asymptomatic cases must be followed up regularly. If a supernumerary kidney is nonfunctional or associated with hydronephrosis, carcinoma, calculi, or pyelonephritis, nephrectomy may be the appropriate procedure (11). In our case, after a thorough history collection, physical examination, laboratory testing, and imaging evaluations, it was found that our patient had no indications for a nephrectomy; therefore, he was discharged with a follow-up appointment.

## 4 Summary

A supernumerary kidney is a rare congenital anomaly. It may exist alone or in combination with other congenital defects. In addition, the patient with this rare condition may also exhibit a series of anomalies, such as hydronephrosis, pyelonephrosis, renal and ureteral calculi, and benign and malignant neoplasms. It may sometimes be an incidental finding. Diagnostic options include MRI, CT urography, intravenous urography, and abdominopelvic ultrasound. With CT urography and MRI, the blood flow to the kidney and gastrointestinal tracts is better visualized. The patient’s condition determines the course of treatment, which can range from conservative treatment to nephrectomy.

## Data Availability

The original contributions presented in the study are included in the article/Supplementary material, further inquiries can be directed to the corresponding author.
