# Supplementary material for: Case report: An incidental finding of a left-sided supernumerary kidney
Source: Front Med (Lausanne). 2025 Jan 17;11:1490211. doi: 10.3389/fmed.2024.1490211 (PMC11783679; doi:10.3389/fmed.2024.1490211)
Supplement: Supplementary file 1 [file Data_Sheet_1.ZIP › Supplementary_Material submission revised.pdf]

Supplementary Material

1.1 Supplementary Figures

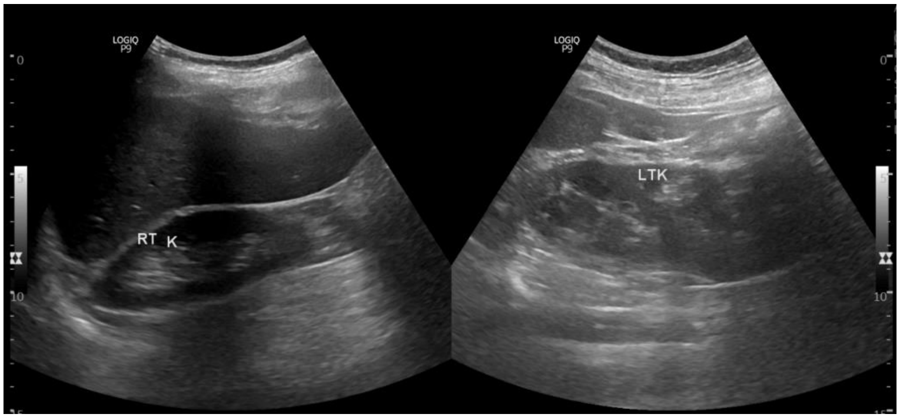

Supplementary Figure 1. Abdominal Ultrasonogram showing right side single kidney and left sided native and supernumerary kidneys

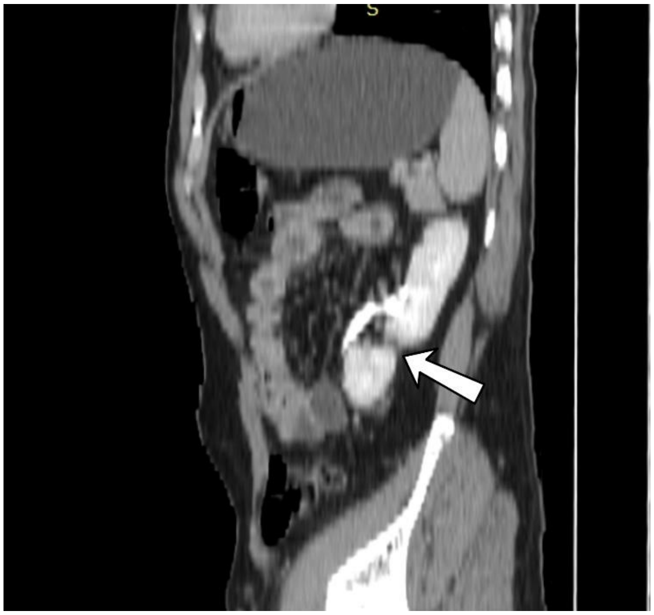

Supplementary Figure 2. Sagittal view of left sided supernumerary kidney on CT urography nephrogenic phase

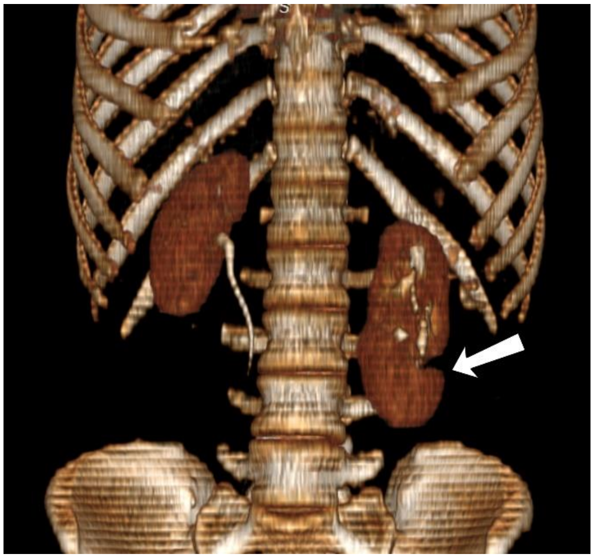

Supplementary Figure 3. 3D reconstruction of the coronal view of CT urography showing left sided native and supernumerary kidneys extending up to L4
