# Supplementary material for: Case report: An incidental finding of a left-sided supernumerary kidney
Source: Front Med (Lausanne). 2025 Jan 17;11:1490211. doi: 10.3389/fmed.2024.1490211 (PMC11783679; doi:10.3389/fmed.2024.1490211)
Supplement: Supplementary file 1 [file Data_Sheet_1.ZIP › Supplementary_Material submission revised.docx]

Supplementary Material

## Supplementary Figures

**
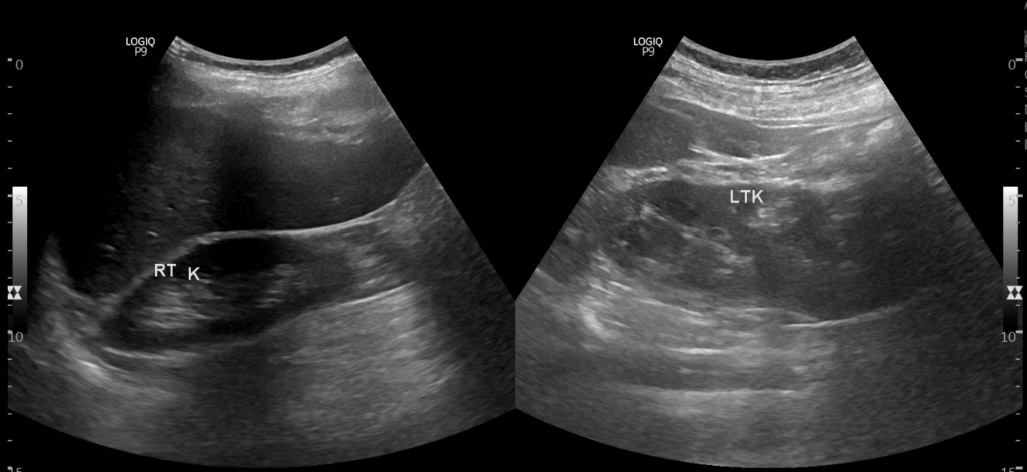
**

**Supplementary Figure 1.** Abdominal Ultrasonogram showing right side single kidney and left sided native and supernumerary kidneys


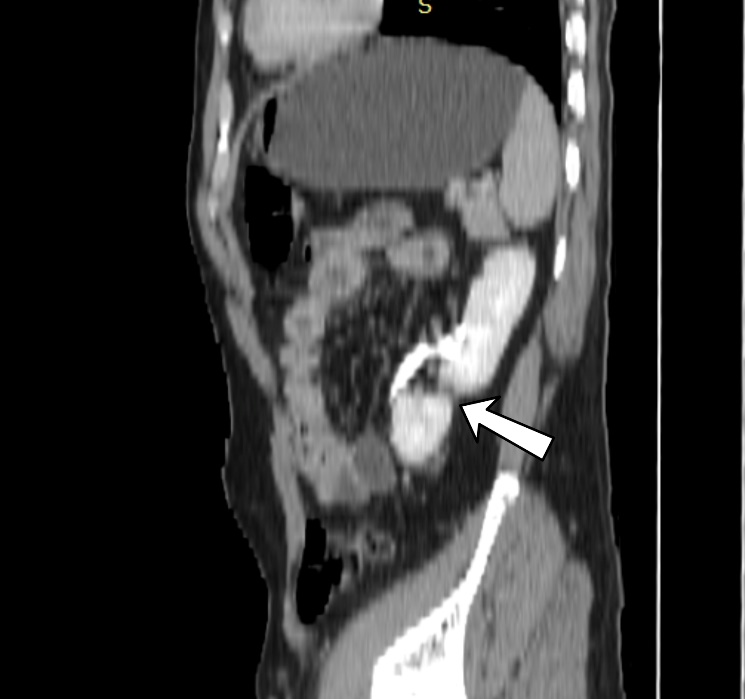


**Supplementary Figure 2.** Sagittal view of left sided supernumerary kidney on CT urography nephrogenic phase


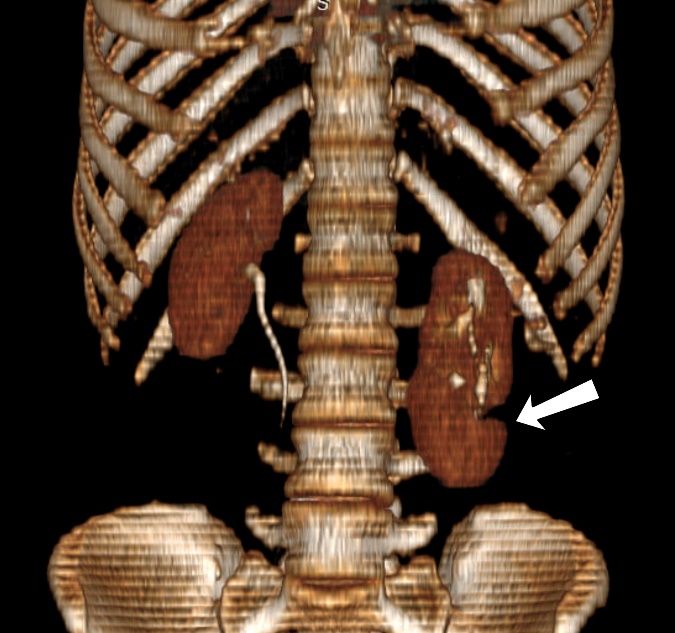


**Supplementary Figure 3.** 3D reconstruction of the coronal view of CT urography showing left sided native and supernumerary kidneys extending up to L4
