# Supplementary figures and images for: Case report: An incidental finding of a left-sided supernumerary kidney
Source: Front Med (Lausanne). 2025 Jan 17;11:1490211. doi: 10.3389/fmed.2024.1490211 (PMC11783679; doi:10.3389/fmed.2024.1490211)

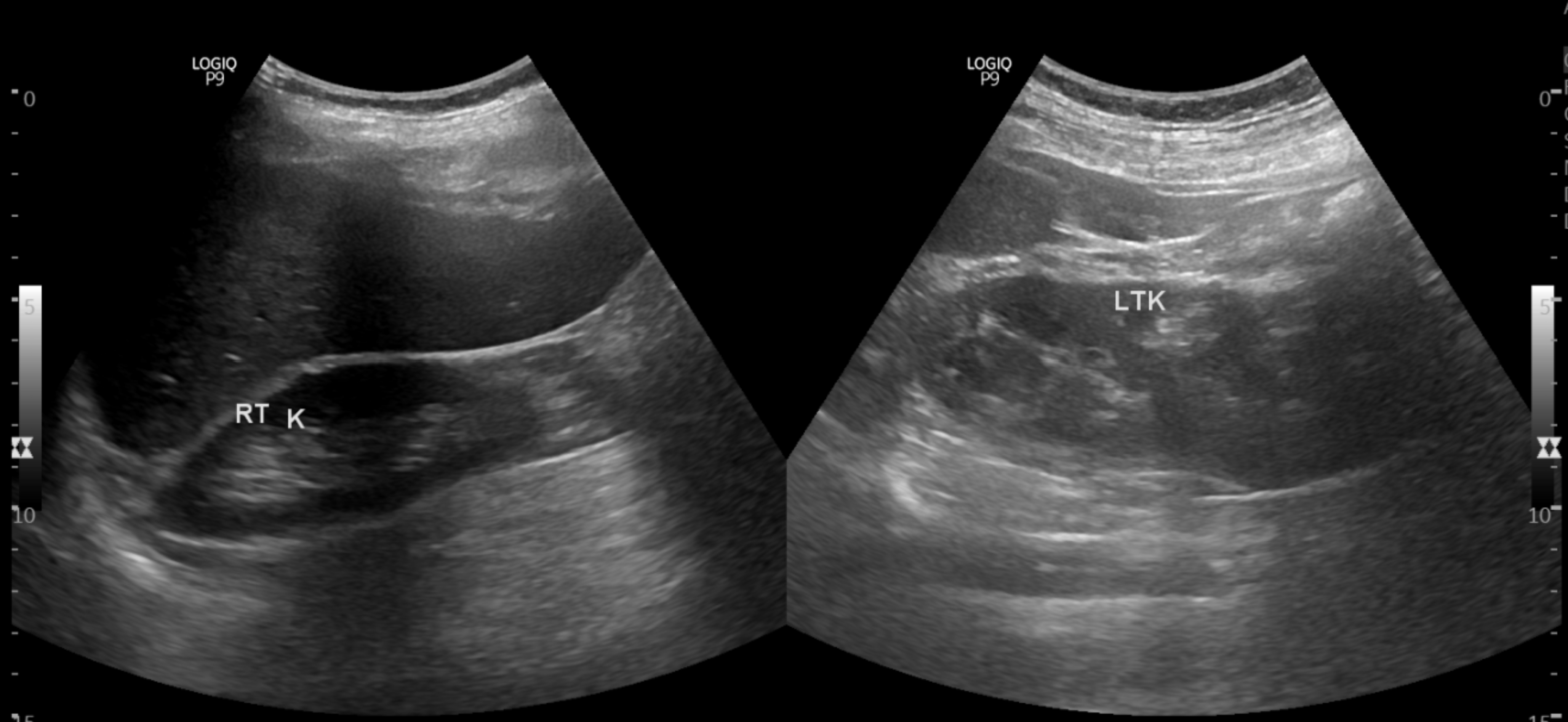

Supplement: Supplementary file 1 [file Data_Sheet_1.ZIP › Figure 1.jpg]

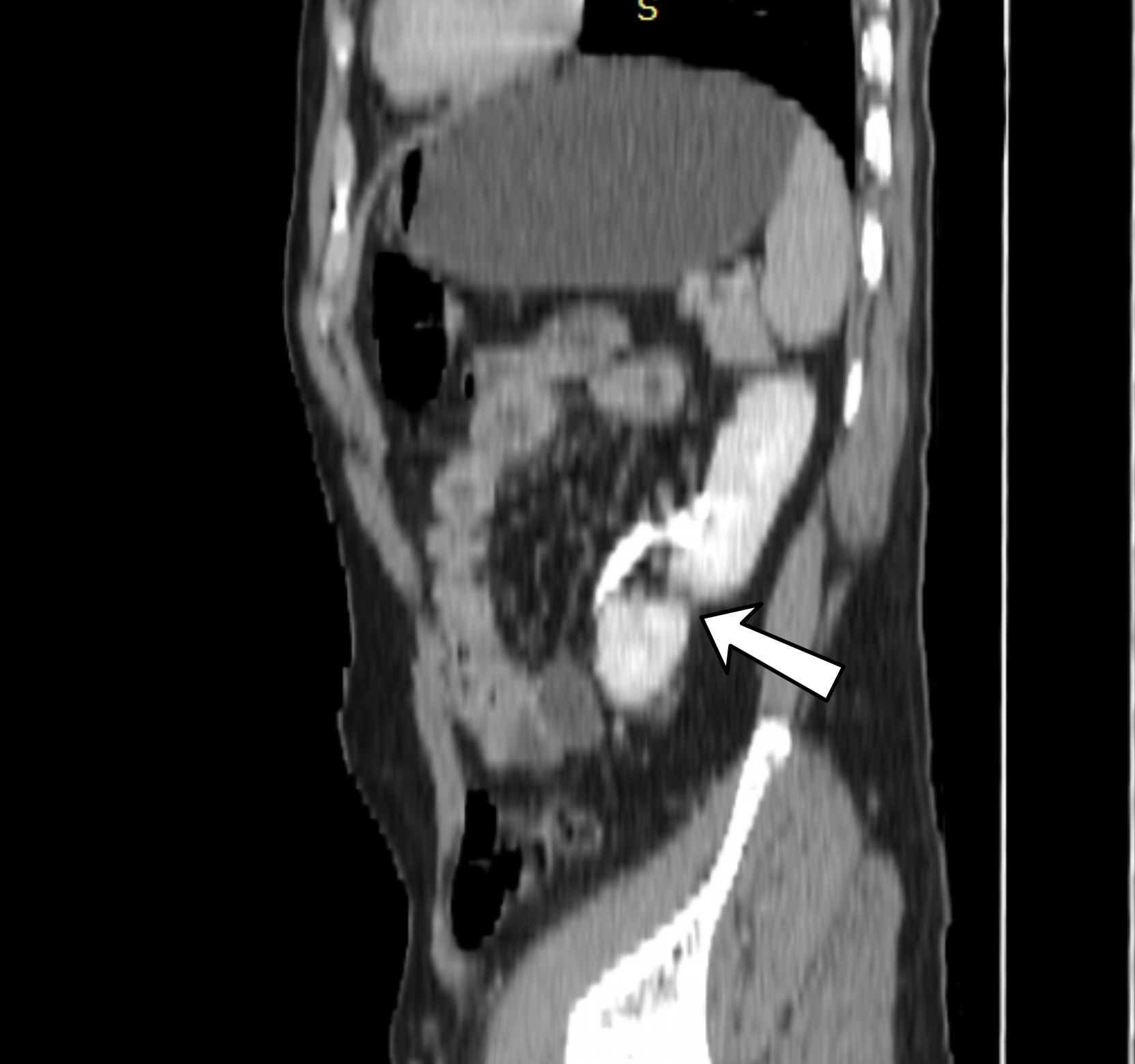

Supplement: Supplementary file 1 [file Data_Sheet_1.ZIP › Figure 2.jpg]

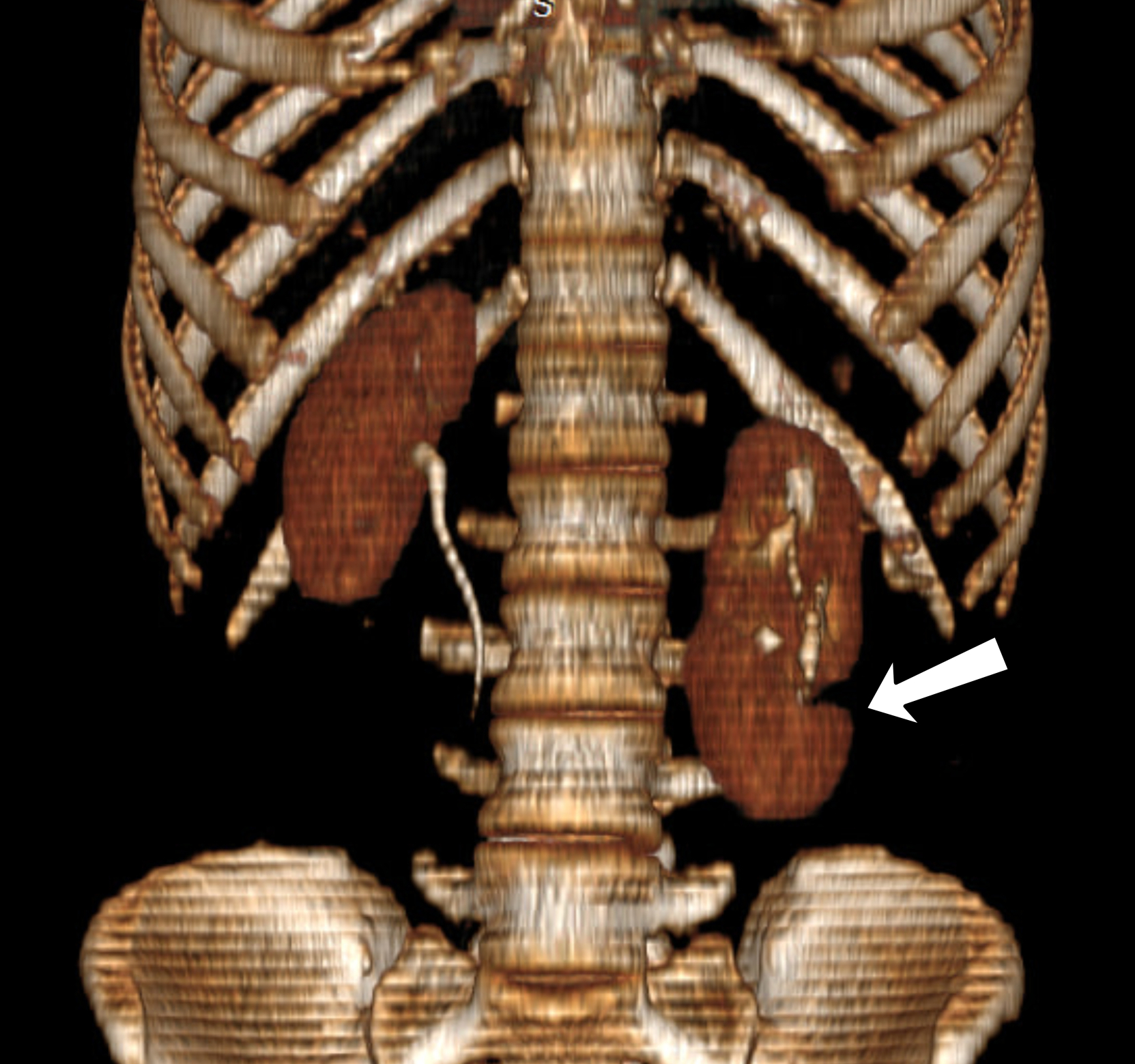

Supplement: Supplementary file 1 [file Data_Sheet_1.ZIP › Figure 3.jpg]
